# Global Membrane Protein Interactome Analysis using *In vivo* Crosslinking and Mass Spectrometry-based Protein Correlation Profiling*[Fn FN2]

**DOI:** 10.1074/mcp.O115.055467

**Published:** 2016-04-25

**Authors:** Mark Larance, Kathryn J. Kirkwood, Michele Tinti, Alejandro Brenes Murillo, Michael A. J. Ferguson, Angus I. Lamond

**Affiliations:** From the ‡Centre for Gene Regulation and Expression, School of Life Sciences, University of Dundee, Dundee, United Kingdom;; §Biological Chemistry and Drug Discovery Division, School of Life Sciences, University of Dundee, Dundee, United Kingdom

## Abstract

We present a methodology using *in vivo* crosslinking combined with HPLC-MS for the global analysis of endogenous protein complexes by protein correlation profiling. Formaldehyde crosslinked protein complexes were extracted with high yield using denaturing buffers that maintained complex solubility during chromatographic separation. We show this efficiently detects both integral membrane and membrane-associated protein complexes,in addition to soluble complexes, allowing identification and analysis of complexes not accessible in native extracts. We compare the protein complexes detected by HPLC-MS protein correlation profiling in both native and formaldehyde crosslinked U2OS cell extracts. These proteome-wide data sets of both *in vivo* crosslinked and native protein complexes from U2OS cells are freely available via a searchable online database (www.peptracker.com/epd). Raw data are also available via ProteomeXchange (identifier PXD003754).

Proteins rarely work as monomers to carry out all the biological processes needed for cells to function. An estimate of the total number of protein-protein interactions within the human proteome, based on currently available data sets, is ∼650,000 (1). This is likely an underestimate, given that many proteins form either transient, or weak interactions within intact cells that may not yet have been detected. This suggests that the majority of human proteins can participate in protein complex formation, at least under some conditions. This includes the many well-studied soluble protein complexes in the cytoplasm, exemplified by the proteasome, ribosomes and cytoskeletal network. It also includes many membrane-associated complexes, for example receptor tyrosine kinase signaling complexes, integrin networks and transmembrane transporters (2). To characterize the many roles of multi-protein complexes in biological regulatory mechanisms, it is important to have convenient methods for the rapid and efficient analysis of their composition and dynamics (3). Ideally, such methods should be applicable to system-wide studies and allow the analysis of endogenous proteins, rather than exclusively use tagged and/or over-expressed baits.

The methods available for the proteome-wide analysis of protein interactions have developed swiftly over the last ten years. This field is dominated by affinity-enrichment based approaches, using either tagged constructs, or antibodies specific for endogenous proteins. Another approach is *in vivo* proximity labeling, based, for example, on the exogenous expression of a protein of interest, fused either to a promiscuous biotin-ligase (BioID) (4), or to a peroxidase enzyme that activates biotin-phenol (APEX) (5). While these data sets have proved very useful, there are some downsides. For example, a large expense in terms of both time and money to generate the thousands of individual “bait” proteins required for global interaction analyses. In addition, each of these affinity enrichments will be performed in only one type of buffer system, which is unlikely to be compatible with the maintenance of all protein-protein interactions. Another dimension to the analytical problem is that many proteins are expressed as different sized isoforms and/or in different post-translationally modified forms, resulting in formation of multiple, related, but functionally distinct complexes, with different combinations of interaction partners (6). Using affinity-enrichment/pull-down methods alone makes it difficult to resolve such mixtures of different forms of related protein complexes, complicating a detailed understanding of biological response mechanisms.

An alternative strategy involves protein correlation profiling-MS, *i.e.* correlating similarities in the fractionation profiles of proteins detected by mass spectrometry, assuming that proteins in a common complex will cofractionate. This approach was previously applied to the analysis of subcellular organelle proteomes (7, 8), and subsequently extended to analyze soluble protein complexes. Thus, recent studies have shown that chromatography-based separation of soluble protein complexes, combined with fraction collection and high-throughput liquid chromatography-tandem mass spectrometry (LC-MS/MS)^1^, facilitates analysis of many hundreds of soluble complexes from a single experiment (6, 9–11). A limitation of all of these studies, however, is that the native extraction conditions used to preserve protein-protein interactions isolates predominantly stable, soluble complexes. For example, many proteins that are integral to membranes are not recovered (12). Similarly, soluble protein complexes that have weakly bound protein subunits can dissociate upon cell lysis and the inevitable dilution associated with extraction. Thus, the potential value of this approach for the system-wide analysis of protein complexes is limited without a covalent tether to hold protein-protein interactions intact during extraction and subsequent chromatographic separation (13).

Covalent protein crosslinking has been used extensively to stabilize protein complexes, cultured cells and tissues for subsequent analysis, either by microscopy, nucleotide sequencing or mass spectrometry. The agents employed to crosslink proteins to each other include various chemical groups able to react with the side-chains of either amino acids, nucleotides, carbohydrates or lipids (14). These crosslinking agents vary in the efficiency with which they perfuse into unbroken cells/tissues and the speed of their reaction when in proximity to a suitable chemical group. One of the most widely used crosslinkers is formaldehyde, which can reversibly form a covalent crosslink to stabilize both protein-protein and protein-nucleotide interactions (15–21). One of the main benefits of using formaldehyde is that because of its small size, it readily permeates intact cells and tissues. Another benefit of using formaldehyde is the easy reversal of the crosslinks by heating and subsequent compatibility with mass spectrometry-based proteome analysis.

Here, we describe a mass spectrometry-based proteomic approach for the efficient global analysis of protein complexes, including membrane proteins, using *in vivo* protein crosslinking combined with denaturing extraction. Using high-resolution, size-exclusion chromatography (SEC) to separate crosslinked complexes under denaturing conditions and MS analysis of fractionated proteins, we could identify membrane bound and membrane associated complexes not accessible in native extracts. We present a detailed comparison of the sets of protein complexes that can be identified using protein correlation profiling MS analysis in conjunction with both formaldehyde crosslinked and native extracts from U2OS cells. We provide access to the entire proteome-wide data sets of both *in vivo* crosslinked and native U2OScell protein complexes via a searchable online database (http://www.peptracker.com/epd/).

## EXPERIMENTAL PROCEDURES

### 

#### 

##### Materials

U2OS cells were purchased from the American Type Culture Collection (ATCC, Rockville, MD). Dulbecco's Modified Eagle Medium (DMEM), doxycycline/tetracycline-free fetal calf serum, antibiotics, NuPage gels, LDS sample buffer, MES SDS-PAGE running buffer, nitrocellulose iBlot stacks, SYPRO Ruby, Alexa Fluor 680-conjugated secondary antibodies, Dulbeccos's Phosphate Buffered Saline (PBS), EZQ protein quantitation reagent and CBQCA assay kit were obtained from Life Technologies (Carlsbad, CA). IrDye 800-conjugated secondary antibodies were obtained from Rockland Immunochemicals (Gilbertsville, PA). HRP conjugated secondaryantibodies were from Cell Signaling Technology (Danvers, MA). Formaldehyde ampules (10 ml, methanol free), bicinchoninic acid (BCA) assay reagents, Coomassie Plus (Bradford) reagent, Detergent Removal Plates, Acclaim Pepmap C18 columns and trapping cartridges and Triscarboxyethylphosphine (TCEP) (Bond-breaker neutral pH solution) were from Thermo Scientific (Waltham, MA). Trypsin Gold was from Promega. Sep-Pak tC18 96-well u-elution plates were from Waters (Milford, MA). GAPDH primary antibody, complete protease inhibitor mixture tablets and PhosStop phosphatase inhibitor tablets were from Roche (Basel, Switzerland). Odyssey Nitrocellulose Membrane was from Li-Cor Biosciences (Lincoln, NE). Ultrafree-MC 0.5 ml, 0.45 μm centrifugal filter units were from Millipore (Billerica, MA). All other materials were obtained from Sigma (St. Louis, MO).

##### Cell Culture

Briefly, U2OS cells were grown in DMEM supplemented with 10% FCS, 100 U/L penicillin and 100 μg/L streptomycin at 37 °C in 10% CO_2_, and passaged at ∼80% confluence.

##### In Vivo Crosslinking and Denaturing Extraction for Size Exclusion Chromatography

Each 15 cm dish (80% confluent) of adherent U2OS cells was washed three times with ice-cold PBS, on ice, with 20 ml used per wash. Plates were drained and 20 ml of freshly made 6% formaldehyde in PBS was added to crosslink proteins and slowly mixed for 30 min at room temperature. After draining, the crosslinked cells were quenched with 20 ml of 0.1 m Tris-HCl pH 8.0, 150 mm NaCl for 10 min at room temperature. After complete drainage of the dish, cells were scraped in 500 μl of freshly prepared lysis buffer (4% SDS, 100 mm NaCl, 10 mm sodium phosphate pH 6.0, 25 mm TCEP, 50 mm N-ethylmaleimide) at room temperature. Cell lysates were sonicated for 30 s, three times in total, at 10% power at room temperature. Lysates were heated to 37 °C for 30 min prior to centrifugation at 17,000 × *g* for 10 min at room temperature. Samples were filtered through 0.45 μm Ultrafree-MC centrifugal filter units.

##### SDS-PAGE and Immunoblotting

For immunoblotting nondenatured samples, Bradford protein quantitation assays were performed on the fractions. 20% SDS was added to each fraction to 2% final concentration and heated to 65 °C for 10 min. 100 μl of consecutive fractions were combined and chloroform methanol precipitation performed (24). Protein was then re-suspended in equal volumes of 1× LDS, 25 mm TCEP so the maximum concentration in the most concentrated fraction was 1 mg/ml, and heated to 65 °C for 10 min. Combined fractions were analyzed by the EZQ quantitation assay. 10 μl of each fraction was loaded per lane for SDS-PAGE. BCA protein quantitation was performed on denatured samples. Equal volumes (14 μl) of consecutive samples were combined and made up to a maximum of 0.1 mg/ml in 1× LDS/TCEP. 20 μl of sample was loaded per lane for SDS-PAGE. SDS-PAGE was performed using 4–12% (w/v) Bis-Tris NuPage gels using MES running buffer according to manufacturer's instructions but with the addition of 25 mm TCEP, in the LDS sample buffer. SYPRO Ruby staining was performed as per manufacturer's instructions. For Western blotting, separated proteins were electrophoretically transferred to either an iBlot nitrocellulose membrane, or Odyssey Nitrocellulose Membrane, blocked with 3% nonfat skim milk in 0.1% Tween-20 in TBS (TBST) and incubated with primary antibody in 5% BSA in TBST overnight at 4 °C. After incubation, membranes were washed three times in TBST and incubated with either HRP labeled, or Alexa fluor 680/IrDye 800 labeled, secondary antibodies in 3% nonfat skim milk in TBST. Proteins were visualized using Immobillon chemiluminescent substrate (Millipore) and imaged, either with a cooled CCD camera (Fuji) for HRP-labeled secondary antibodies, or a Licor Odyssey CLx imager for Alexa fluor 680/IrDye 800 labeled secondary antibodies.

##### Denaturing Size Exclusion Chromatography, Protein Digestion, and Peptide Clean-up

Using a Dionex Ultimate 3000 Bio-RS UHPLC system (Thermo Scientific), lysates were injected (100 μl per injection) onto a BioBasic SEC1000 column (300 × 7.8 mm, 5 μm particles, 1000Å pores) equilibrated with 0.2% SDS, 100 mm NaCl and 10 mm NaPO_4_ pH 6.0 at 30 °C. A buffer at pH 6.0 is used to prolong column lifetime. The flow rate was 0.2 ml min^−1^ and for each sample two injections were performed. For each injection 48 × 125 μl fractions were collected separately using a 96-well thin-walled PCR plate (Eppendorf) and heated to 95 °C for 30 min in a PCR machine, using a heated lid, to break all crosslinks. After heat reversal of crosslinks, samples were transferred to a 96-well low protein binding deep-well plate (Eppendorf) and Tris-HCl (1 m pH 8.0) was added to each fraction to a final concentration of 0.1 m to adjust the pH to 8.0. Proteins in each fraction were digested to peptides using either LysC alone (injection 1), or LysC and trypsin (injection 2), which were diluted in 0.1 m Tris-HCl and added at a ratio of 1:50 by weight, based upon an EZQ protein assay of the fractions, then incubated for 18 h at 37 °C.

##### Peptide Clean-up and Quantitation

SDS in peptide samples was removed using 96-well detergent removal plates (Thermo Scientific) and centrifugation according to manufacturer's instructions. Briefly, the resin in each well was washed three times with 300 μl of room temperature PBS, with centrifugation at 1000 × *g* to remove the solution after each wash. Peptide samples were applied to the resin in each well and incubated for 2 min at room temperature before collecting the filtrate (containing clean peptides) by centrifugation for 2 min at 1,000 g at room temperature into a 96-well low protein binding deepwell plate (Eppendorf). Peptides were then desalted after trifluoroacetic acid (TFA) was added to 1% (v/v) final concentration and peptides were purified using a Sep-Pak tC18 96-well u-elution plate (Waters). Peptides were eluted in 200 μl of 50% (v/v) acetonitrile 0.1% TFA and evaporated to dryness in a rotary evaporator prior to re-suspension in 5% (v/v) formic acid. Peptide concentrations were determined using the CBQCA assay (Thermo Scientific) and peptide standards derived from a BSA digest, after 25-fold dilution of peptide samples in 0.1 m borate buffer pH 9.3.

##### LC-MS/MS and Analysis of Spectra

Using a Thermo Fisher Scientific Ultimate 3000 RSLCnano UHPLC, peptides in 5% (v/v) formic acid (final volume ∼10 μl) were injected onto an Acclaim PepMap C18 nano-trap column. After washing with 2% (v/v) acetonitrile, 0.1% (v/v) formic acid, peptides were resolved on a 50 cm × 75 μm C18 EasySpray reverse phase analytical column with integrated emitter over a gradient from 2% acetonitrile to 35% acetonitrile over 220 min with a flow rate of 200 nL min-1. The peptides were ionized by electrospray ionization at +2.0 kV. Tandem mass spectrometry analysis was carried out on a Q-Exactive mass spectrometer (Thermo Fisher Scientific) using HCD fragmentation. The data-dependent acquisition method used acquired MS/MS spectra on the top 30 most abundant ions at any one point during the gradient. All of the RAW MS data have been deposited to the ProteomeXchange Consortium (http://proteomecentral.proteomexchange.org) via the PRIDE partner repository with the data set identifier PXD003754. The RAW data produced by the mass spectrometer were analyzed using the MaxQuant quantitative proteomics software package (22) (http://www.maxquant.org, version 1.5.1.3). The MaxQuant output has also been uploaded to the ProteomeXchange Consortium under the same identifier given above. This version of MaxQuant includes an integrated search engine, Andromeda (23). Peptide and Protein level identification were both set to a false discovery rate of 1% using a target-decoy based strategy. The database supplied to the search engine for peptide identifications was the Human Swissprot database downloaded on the April 17, 2015, containing 20,197 protein sequence entries. The mass tolerance was set to 4.5 ppm for precursor ions and MS/MS mass tolerance was set at 20 ppm. Enzyme was set to either LysC (cleavage C-terminal to lysine) or trypsin (cleavage C-terminal to lysine and arginine) with up to 2 missed cleavages. Deamidation of Asn and Gln, oxidation of Met, pyro-Glu (with peptide N-term Gln), phosphorylation of Ser/Thr/Tyr, and protein N-terminal acetylation were set as variable modifications. N-ethylmaleimide on Cys was searched as a fixed modification. The output from MaxQuant provided peptide level data as well as protein group level data. We used the protein groups as defined by the Maxquant package (22).

##### Experimental Design and Statistical Rationale

Three biological replicates were performed for the *in vivo* crosslinking and denaturing SEC analysis and this level of replication was chosen based upon the variance detected in previous experiments using SEC-based analysis (6). To achieve an unbiased analysis of native *versus* crosslinked fractions, we performed a combined MaxQuant analysis of RAW files from our previous U2OS Native SEC analysis (6) and the PFA Crosslinked U2OS SEC analysis described here. To create an elution profile for an individual protein group in each of the three biological replicates in each experiment type (either native, or PFA crosslinked), we used the MaxQuant label free quantitation (LFQ) algorithm (24).

##### Initial Data Processing and Basic Clustering Analysis

These steps were performed using the R language (version 3.2.2). The LFQ intensity profile for each replicate was smoothed using a three-fraction sliding mean and the minima and maxima of each profile was normalized within the limits 0 and 1 respectively. The mean and standard deviation for each protein in each experiment type (*i.e.* either native, or crosslinked) across three biological replicates was calculated for subsequent plotting using the ggplot2 package (http://ggplot2.org/), correlation analysis, basic clustering and the machine learning-based protein complex prediction. From the three biological replicates, it was required that a protein be identified in at least two out of three replicates, with a minimum of two peptides in each. Proteins labeled as either contaminants, or reverse hits, were removed from the analysis. The mean profiles for each protein were hierarchically clustered within each experiment type (either native, or PFA-crosslinked). The basic hierarchical clustering was performed, separately, for the respective native and crosslinked data sets, using the Euclidean distance measurement and a 'complete' agglomeration method. The tree calculated for each data set was cut to generate clusters with a mean Pearson correlation coefficient of ∼0.95.

##### Comparison of Known Complexes between Native and Crosslinked Extracts

We compared each of the previously annotated protein complexes, either from CORUM (25), or from the most recent analysis of human cells with PCP-based analysis (10), with both our native and formaldehyde crosslinked U2OS cell protein data sets. For each protein complex we determined the number of member proteins identified in either our native, or crosslinked data sets. A protein complex was analyzed if the number of identified protein subunits was greater than or equal to 2 in either the native, or crosslinked data sets. For these selected protein complexes we determined the median Pearson correlation coefficient between all the possible combinations of unique protein pairs in the complex.

##### Machine Learning-based Protein Complex Prediction

We applied a pipeline similar to that applied previously for PCP analysis (9, 10) to predict protein complexes from the crosslinked data set. First, we compiled a custom python script to extract peaks from the LFQ intensity profiles. We used the scipy package (26) to adapt a Ricker wavelet encompassing 2 to 8 fractions. The two minimum points of the wavelet were used to define the peak range. Any other profile values outside this range were set to 0. We applied several filters before considering peaks further. First, we set an arbitrary noise threshold for each peak at 15% of the maximum signal intensity of the profile. Second, we discarded peaks lying in a region of the fractionation profile corresponding to predicted molecular weight values either approximately equal to, or below, the protein dimer molecular weight. Third, we discarded peaks present in the void region (fractions 1 to 4). Fourth, we only considered peaks whose maxima were separated by a minimum of 3 fractions.

The resulting peak profiles were used to identify protein complexes using a machine learning approach (9, 10). We used a logistic regression implemented with the scikit-learn python package (27) to score peak pairs according to 6 features, namely: Coapex, Normalized Cross Correlation (NCC), Pearson Correlation Coefficient (PCC), String Score, HIPPIE score and Mentha score. The first three features are purely based on the peak profiles. The Coapex was used by Havugimana *et al.* (9) and is based on the number of experiments (replicates in the crosslinked data set) in which the peak pairs showed maximum abundance in the same peak fraction. For our data set of 3 biological replicates the possible coapex scores were: 1 (3 of 3 replicates), 0.6 (2 of 3 replicates), 0.3 (1 of 3 replicates), and 0 (none of the replicates). The NCC was derived in 2 steps. First, we computed the maximum cross correlation between the two peak pairs P_1–2_CC. We then computed the maximum self-cross-correlation of the first peak (P_1_CC) and the max self-cross-correlation of the second peak (P_2_CC). The NCC was finally derived as P_1–2_CC/max(P_1_CC, P_2_CC). The NCC assume values between 0 and 1. The PCC was computed as the Pearson correlation score between the two peaks and range in value from −1 to 1. The other 3 features are derived from protein interaction databases, as described previously (9, 10). The rationale for these features is to try to promote peak pairs from proteins that have been previously reported to interact in the literature. With this we attempted to de-noise the data from peaks that may have similar elution profiles by chance. The String score was taken from the STRING database (version 10) (28) and was normalized to have values from 0 to 1. The mentha score was taken from the mentha database (version 06–12-2015) (29) and the hippie score from the HIPPIE database (version 09–01-15). Both the menthe and the hippie scores ranged in value from 0 to 1. We calculated the 6 features for all the possible permutations of peak pairs that showed the maximum abundance in the same fraction ± 1, creating a matrix (test set) of 1,394,292 peak pairs each with six features.

For the machine learning we first assembled a data set of “gold standard” true positive peak pairs (GD). To obtain these pairs we used the CORUM database of curated protein complexes and extracted all the peak pairs belonging to 90 complexes, creating a matrix of 551 unique true positive peak pairs. A true negative data set was extracted by random sampling of the 551 true positive peak pairs, between all the possible combinations of peaks belonging to proteins annotated in different complexes. As it would be possible to introduce false negative interactions in this step, we repeated the random sampling 100 times. Finally, using these true positive and true negative test pairs we assembled 100 logistic regression classifiers based on the same true positive pairs, but with each using a different true negative set. All the classifiers were inspected to determine the AUC values of the ROC curve in 10-fold cross validation. The median values of the probability score outputs of the 100 classifiers were used as the final score for the test set. We selected a score cut-off of 0.75 and we imported the 52,048 peak pairs above this threshold to the ClusterONE algorithm (30). We created a search matrix for the ClusterONE program with the parameters d (0.1 to 1, step 0.1), haircut (0.1 to 1, step 0.1) and s fixed to 2. The output was parsed to derive the parameters that were optimal to obtain the maximum number of GD true positive peaks grouped together.

## RESULTS

**Fig. 1.**
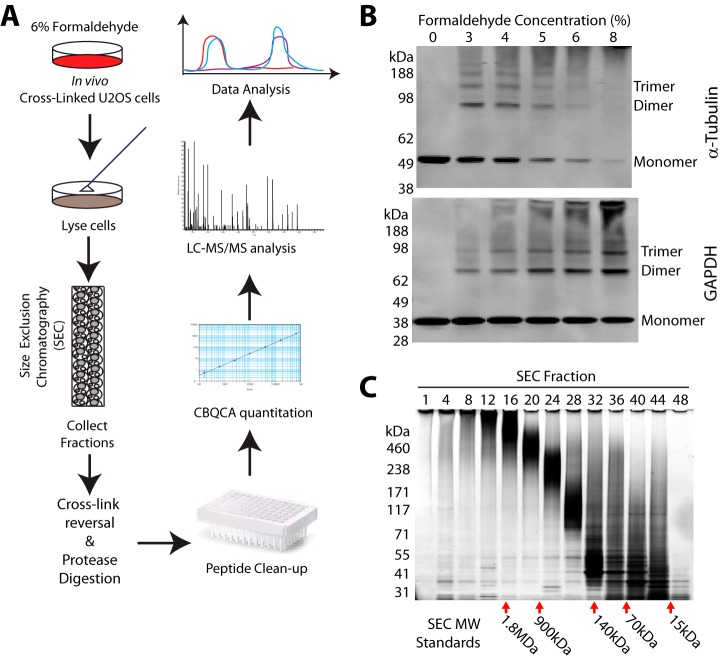
**Stabilization of protein interactions by *in vivo* crosslinking enables separation of protein complexes.** *A*, Workflow for the cellular crosslinking, complex extraction and LC-MS/MS methodology. *B*, Titration of crosslinker concentration applied to U2OS cells and immunoblot analysis of total cell lysates, to determine optimal crosslinking conditions. Complexes with known subunit structure were used and the known multimeric structure of each band is indicated on the right of each blot (*n* = 3). *C*, SDS-PAGE analysis of crosslinked U2OS cells separated by denaturing-SEC, demonstrates intact complexes migrate at smaller apparent molecular weights compared with the linear standards used for SDS-PAGE (shown on the left of the gel image) (*n* = 3).

To improve the efficiency of chromatography-MS based global analyses of protein complexes and circumvent the under-representation of membrane complexes and complexes tightly bound to cell substructures, we have developed a methodology that combines *in vivo* protein crosslinking prior to cell lysis with subsequent SEC fractionation and MS analysis (Fig. 1*A*). By first covalently locking protein-protein interactions in place *in vivo*, it is possible to maximize the efficiency of protein recovery using highly denaturing buffer conditions to solubilize essentially all complexes in the cell extract. We have evaluated this approach using the human U2OS osteosarcoma cell line, which is widely used by cell biologists for the study of cellular response mechanisms.

We employed formaldehyde as the *in vivo* crosslinker, exploiting its known fast and efficient cell penetration and previous successful application as a crosslinker in immunofluorescence microscopy and chromatin immunoprecipitation methods. To determine a suitable amount of formaldehyde for use in U2OS cells, we first titrated the concentration applied to intact, adherent U2OS cells, aiming for a concentration of formaldehyde that resulted in isolation of tubulin as predominantly multimers *i.e.* larger than dimers, while simultaneously recovering GAPDH predominantly in complexes not larger than tetramers (Fig. 1*B*). After quenching the reaction with Tris buffer, cellular proteins were extracted in SDS denaturing buffer for immunoblotting. This analysis of crosslinked lysates showed that 6% formaldehyde gave optimal results, as judged by recovery of the highest proportion of large multimers and tetramers, respectively, for the marker proteins tubulin-a1 and GAPDH (Fig. 1*B*).

For large-scale separation of crosslinked protein complexes, HPLC size-exclusion chromatography (SEC) was used. To maximize protein extraction, denaturing cell lysis was performed with 4% SDS under reducing conditions. Clarified lysates were injected onto a high-resolution, silica-based SEC column with 1000 Ångstrom pores, allowing separation of complexes >2MDa. The SEC separation was performed in the presence of 0.2% SDS, which is below the detergent's critical micelle concentration and maintained protein solubility during separation. SDS-PAGE analysis of the 48 SEC fractions collected, together with analysis of marker complexes, show this achieves an effective separation range spanning average molecular weights from >1.8MDa, down to ∼8kDa (Fig. 1*C*). We also confirmed that the crosslinking maintained the integrity of higher molecular weight complexes during the SDS denaturing extraction and SEC workflow, as shown by comparison of SEC chromatograms and SDS-PAGE analysis of SEC fractions derived from cells either crosslinked with 6% formaldehyde, or not crosslinked (supplemental Fig. S1).

Under these optimized conditions, three biological replicates were performed for the systematic analysis of U2OS cell protein complexes, after crosslinking *in vivo* with 6% formaldehyde (Fig. 1*A*). Each fraction was digested in the presence of SDS, either with LysC alone, or a combination of LysC and trypsin, to improve sequence coverage (31). Peptides from each SEC fraction were cleaned to remove the SDS and salts prior to LC-MS/MS analysis on a QExactive mass spectrometer. To facilitate comparison between this crosslinking-based workflow and the analysis of native complexes from the same U2OS cell line, we simultaneously analyzed using MaxQuant the crosslinked SEC data set and an SEC data set generated for native protein complexes (6). Together, these data yielded >120,000 unique peptides detected across all three replicates (supplemental Table S1). These were aggregated to form protein groups (supplemental Table S2), which were subsequently filtered to >4600 protein groups with ≥2 peptides detected in at least two out of three biological replicates for each data set (supplemental Table S2). Normalized LFQ Intensities were calculated separately for each protein group within each SEC fraction in each experiment type (either native, or crosslinked).

**Fig. 2.**
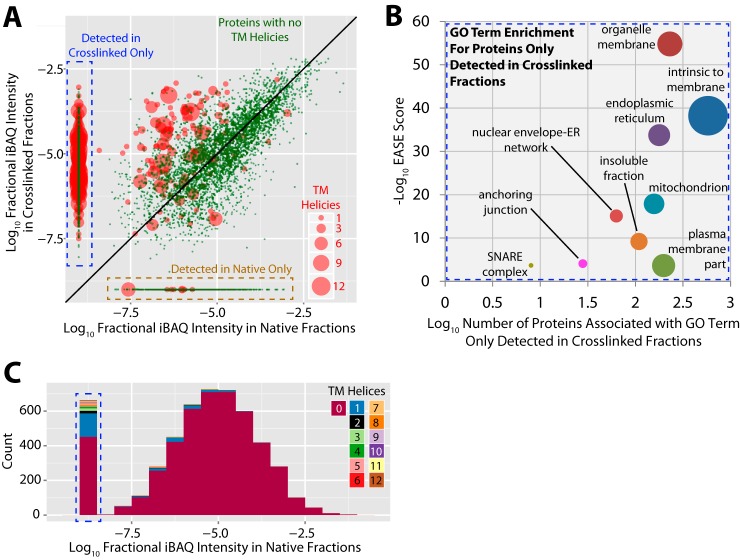
**Integral membrane protein detection is enabled by *in vivo* crosslinking and denaturing extraction.** *A*, The fractional intensity of each protein detected (intensity of each protein/sum of all intensity in data set) in either a native protein complex extraction (6), or the *in vivo* crosslinked and denatured extract from this study, are plotted. Proteins detected in either only the native extracts (dashed yellow box), or only the crosslinked and denatured extracts (dashed blue box), are also shown. Proteins without predicted transmembrane (TM) helices are shown as green dots. Proteins with one or more TM helices are shown as red circles, with the size of each circle being proportional to the number of TM helices in each protein. The diagonal line indicates equal fractional intensity between data sets. A representative experiment is shown (*n* = 3). *B*, Analysis of enriched GO terms (cellular component) within the proteins detected only from crosslinked and denatured extracts. The *y* axis indicates the EASE score (-log_10_ transformed) for each GO cellular component term enriched data set *versus* the whole human proteome, all terms have a *p* < 0.001. The *x* axis shows the number of proteins detected only from crosslinked and denatured extracts associated with each GO term. The size of the circle representing each GO term increases with higher proportional contribution to the subset of proteins detected only from crosslinked and denatured extracts. Colors are randomly chosen. A representative experiment is shown (*n* = 3). *C*, Stacked histogram showing the distribution of proteins with various numbers of TM helices from none up to 12 (indicated in colored legend) across the fractional intensity range from native protein complex extraction (6). Data from proteins only detected in the crosslinked and denatured extracts (dashed blue box) are also shown. A representative experiment is shown (*n* = 3).

To compare the efficiency of this analytical method with our previous analyses of U2OS soluble protein complexes (6), we first determined the relative abundances of each protein between the native and crosslinked workflows. To ensure we were only analyzing well-resolved proteins and not proteins present in unresolved void fractions from the SEC column, the void fractions were removed from the analysis for the crosslinked and native data sets. The fractional iBAQ intensity for each protein was calculated by dividing the individual iBAQ intensity of each protein by the sum of the iBAQ intensities for all proteins in the same data set. In addition, proteins were divided into two groups, *i.e.* those that contain predicted transmembrane helices, as judged using the TMHMM package (32), and those that do not. These fractional iBAQ values were then compared between the two data sets, which showed a strong correlation (>0.8) for those proteins with no predicted transmembrane helices (Fig. 2*A*). However, most (∼600) proteins that contained transmembrane helices were only detected in the crosslinked sample (Fig. 2*A*, transmembrane proteins highlighted in red). Furthermore, those proteins with transmembrane helices detected in both data sets were much more abundant in the crosslinked samples, compared with the native extracts. A gene ontology cellular component enrichment analysis showed that the ∼1000 proteins detected exclusively in the crosslinked data set were mainly from organelle membranes, mitochondria, endoplasmic reticulum, plasma membrane, and insoluble fractions (Fig. 2*B*). Strikingly, further analysis showed that most proteins with more than one transmembrane helix were only detected in the crosslinked samples (Fig. 2*C*).

**Fig. 3.**
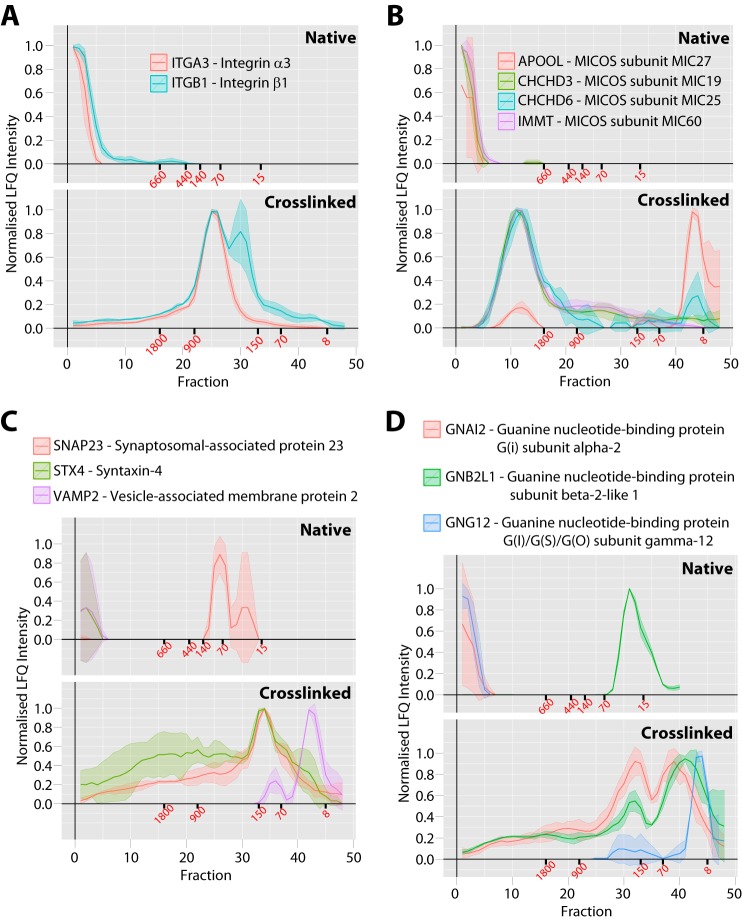
**Comparison between native and crosslinked SEC workflows for membrane protein complex analysis.** *A*, Plasma membrane integrin alpha3-beta1 complex, *B*, mitochondrial inner membrane MICOS complex, *C*, exocytosis STX4-SNAP23-VAMP2 SNARE complex, *D*, plasma membrane GNAI2-GNB2L1-GNG12 G-protein complex. For each complex the upper gray panel shows the protein profiles from the previous native SEC method (6) with 40 fractions total and the lower gray panel shows the profiles from the *in vivo* crosslinked and denatured extract from this study with 48 fractions total. The *x* axis shows the fraction number and the *y* axis shows the normalized LFQ intensity. The line is the mean profile and the surrounding ribbon shows the standard deviation across the three biological replicates (*n* = 3). The elution points for molecular weight standards are shown in red text under each axis in kDa.

To analyze more directly the performance of the entire crosslinking SEC workflow compared with our previous protein correlation profiling analysis of complexes identified in native extracts, we plotted the elution profiles for a series of well-known complexes. Initially, we focused on four integral membrane complexes that were likely to be difficult to analyze using nondetergent based methods. First, we examined the plasma membrane localized integrin α3-β1 heterodimer complex (Fig 3*A*), with each of these proteins containing a transmembrane domain, a series of N-glycan modifications and alpha3 is also palmitoylated (33–36). Each of these proteins was only detected in void fractions under native conditions. However, in the crosslinked data set, the peaks for the same proteins are resolved and overlap, with the beta1 integrin also showing a smaller peak not coeluting with alpha3. Second, the mitochondrial inner membrane localized MICOS complex (37) was not resolved under native conditions, but a clear coeluting peak is detected at ∼2 MDa with the crosslinked method (Fig 3*B*). Third, we examined the SNARE proteins involved in vesicle trafficking to the plasma membrane (38), with VAMP2 and STX4 containing transmembrane domains and SNAP23 having a C-terminal lipid modification to allow membrane association (Fig 3*C*). Under native conditions VAMP2 and STX4 were only detected in void fractions and not well resolved, with only SNAP23 showing clear resolution. In contrast, using the crosslinking workflow we observed a clear coeluting peak of STX4 and SNAP23 at ∼150 kDa with VAMP2 only detected in smaller fractions, which is consistent with previous data showing that STX4 and SNAP23 form a stable complex prior to transient ternary complex formation with VAMP2 to facilitate membrane fusion. Fourth, we examined the hetero-trimeric G-protein complex composed of an α, β, and γ subunit (Fig 3*D*) (39). Using the native workflow only the beta subunit was resolved, which eluted as a single peak at its monomeric MW. Conversely, using the crosslinking method both the alpha and beta subunits coeluted in a clear peak at ∼200 kDa, with the gamma subunit also showing a peak in this size region.

**Fig. 4.**
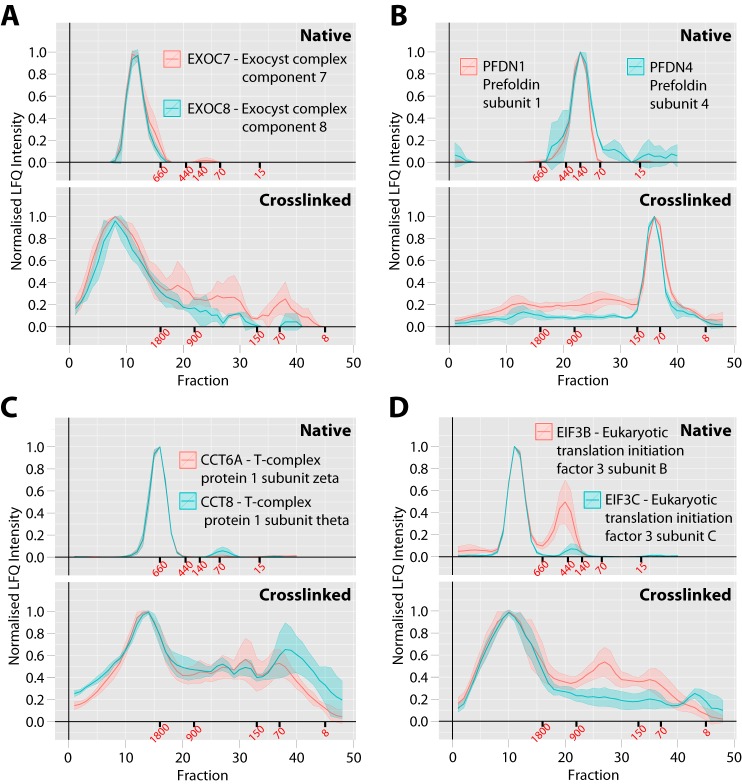
**Comparison between native and crosslinked SEC workflows for soluble protein complex analysis.** *A*, Exocyst vesicle tethering complex, *B*, prefoldin chaperone complex, *C*, TRiC/CCT chaperone complex, *D*, EIF3 translation initiation factor complex. For each complex the upper gray panel shows the protein profiles from the previous native SEC method (6) with 40 fractions total and the lower gray panel shows the profiles from the *in vivo* crosslinked and denatured extract from this study with 48 fractions total. The *x* axis shows the fraction number and the *y* axis shows the normalized LFQ intensity. The line is the mean profile and the surrounding ribbon shows the standard deviation across the three biological replicates (*n* = 3). The elution points for molecular weight standards are shown in red text under each axis in kDa.

We observed many complexes that could be resolved as distinct peaks in both the native and the crosslinked workflows. Most of these complexes were soluble cytosolic complexes, for example including the exocyst complex (Fig 4*A*), the prefoldin complex (Fig 4*B*), the T-complex (Fig 4*C*) and the EIF3 complex (Fig 4*D*). There were some differences between the native and crosslinked profiles for each of these complexes. For example, the chaperone T-complex resolved as a single peak under native conditions was detected with multiple peaks under crosslinked conditions. This includes one main peak >1.8 MDa and several smaller peaks between 900–200 kDa.

There were also several complexes that appeared to be well resolved under native conditions, but either had a complicated elution profile, or else did not elute as an intact complex with the crosslinked method. First, the DNA replication and licensing MCM complex was observed as a single peak under native conditions, but showed multiple overlapped peaks with the crosslinked method, possibly because of the crosslinking of this complex with DNA and chromatin complexes of different sizes (supplemental Fig. S2*A*). A similar outcome was observed for the U2-snRNP, which is a subunit of the RNA splicing machinery (supplemental Fig. S2*B*). The HSP90 chaperone complex was observed as a single peak at ∼500 kDa under native conditions and a peak of similar size was also observed with the crosslinked method. However, in the crosslinked data set the HSP90 complex was also observed in a broad elution pattern at larger sizes, likely representing detection of this chaperone interacting with diverse substrates (supplemental Fig. S2*C*). Interestingly, the mitochondrial ribosome 28S subunit was well resolved under native conditions, but the constituent proteins were only detected at their monomeric molecular weights with the crosslinked method (supplemental Fig. S2*D*). Similarly the proteins belonging to mitochondrial Complex I of the respiratory chain were largely detected only at their monomeric molecular weights with the crosslinking method (supplemental Fig. S3). This suggests that these complexes present in the mitochondrial matrix had dissociated under the denaturing lysis and SEC conditions used here, likely because they were not crosslinked adequately with the crosslinker concentration/type used.

**Fig. 5.**
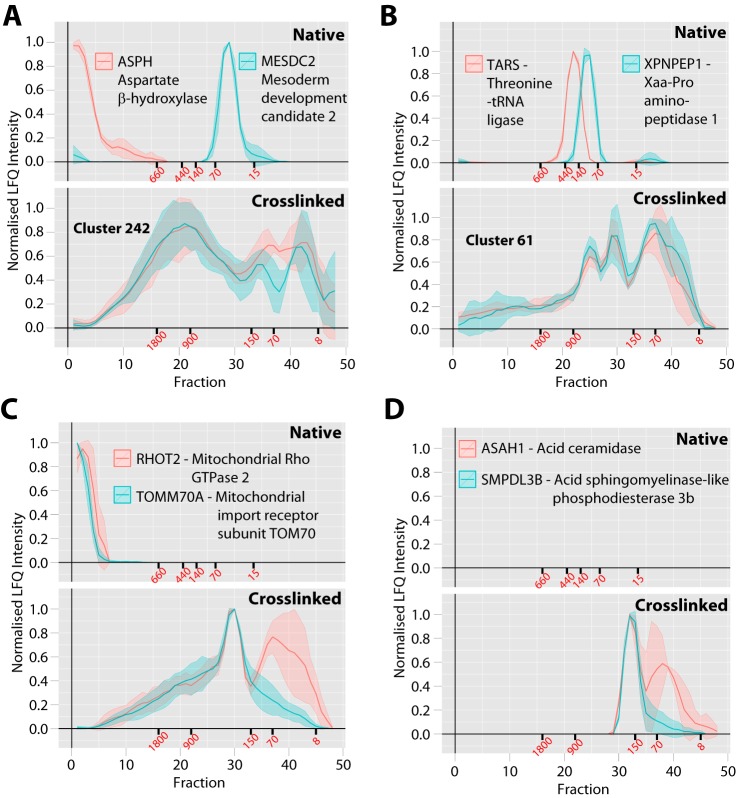
**Comparison between native and crosslinked SEC workflows for novel membrane protein interactions.** *A*, Endoplasmic reticulum ASPH-MESDC2 EGF-like domain chaperone complex, *B*, TARS-XPNPEP1 complex, *C*, Mitochondrial outer membrane TOMM70A-RHOT2 complex, *D*, lysosomal/plasma membrane ASAH1-SMPDL3B sphingomyelin degradation complex. For each complex the upper gray panel shows the protein profiles from the previous native SEC method (6) with 40 fractions total and the lower gray panel shows the profiles from the *in vivo* crosslinked and denatured extract from this study with 48 fractions total. The *x* axis shows the fraction number and the *y* axis shows the normalized LFQ intensity. The line is the mean profile and the surrounding ribbon shows the standard deviation across the three biological replicates (*n* = 3). If coclustered by the basic whole profile clustering analysis, the cluster number is indicated. The elution points for molecular weight standards are shown in red text under each axis in kDa.

To start the identification of previously unknown protein-protein interactions, the profiles were hierarchically clustered, as previously described (6), to identify highly similar whole SEC profiles (Pearson correlation ∼0.95) between different protein groups (supplemental Table S2). This clustering analysis provides predictions that proteins may interact to form common complexes on the basis of the coelution across their entire profile. It also shows where previously proposed interactions are not detected in U2OS cells, *i.e.* when there is no overlap in their respective elution profiles. Using these clusters and also manual curation of the profiles, we observed a number of highly similar profile pairs. First, we observed aspartate beta-hydroxylase (ASPH) and mesoderm development candidate 2 (MESDC2), both of which are ER-resident proteins with their functional domains located in the ER lumen, coclustering in the crosslinked data set (Fig 5*A*) (40–43). Both of these proteins are critical for the folding specifically needed for EGF-like domains present in several hundred human proteins (40–43), including the extracellular regions of some cell surface receptors. In the native data set coelution of the two proteins was not observed, with ASPH only detected in the void fractions, likely because it contains a transmembrane domain, and the soluble MESDC2 protein was detected at its monomeric size. This suggests that the interaction between ASPH and MESDC2 is weak and needs to be stabilized by crosslinks to be observed. A similar pattern was observed for the threonine-tRNA ligase (TARS) and Xaa-Pro aminopeptidase 1 (XPNPEP1), which was coclustered in the crosslinked data set but did not coelute in the native data set (Fig 5*B*). A previous study identified an interaction between XPNPEP1 and several other tRNA ligases (10). However, to our knowledge, an interaction between TARS and XPNPEP1 has not been reported previously.

Two further examples of novel protein-protein interactions observed by coelution of a single peak within multi-peak profiles of the crosslinked data set are highlighted. First, mitochondrial Rho GTPase 2 (RHOT2) and the mitochondrial import receptor subunit (TOMM70A) coeluted in the crosslinked data set at ∼250 kDa (Fig 5*C*), with each of these proteins known to be present on the outer leaflet of the mitochondrial outer membrane (44, 45). Both RHOT2 and TOMM70A were only observed in void fractions in the native separation. Second, acid ceramidase (ASAH1) and acid-sphingomyelinase-like phosphodiesterase 3b (SMPDL3B), coeluted in the crosslinked data set at ∼150 kDa (Fig 5*D*), with each of these proteins known to be present in either the lysosomal lumen, or plasma membrane and catalyzing consecutive steps in the conversion of sphingomyelin to sphingosine (46, 47). Supporting evidence for this interactionis provided by previously reported binding between the SMPDL3B-paralog sphingomyelin phosphodiesterase 1 (SMPD1)and ASAH1 (48). Neither ASAH1, nor SMPDL3B, were detected in the native data set.

**Fig. 6.**
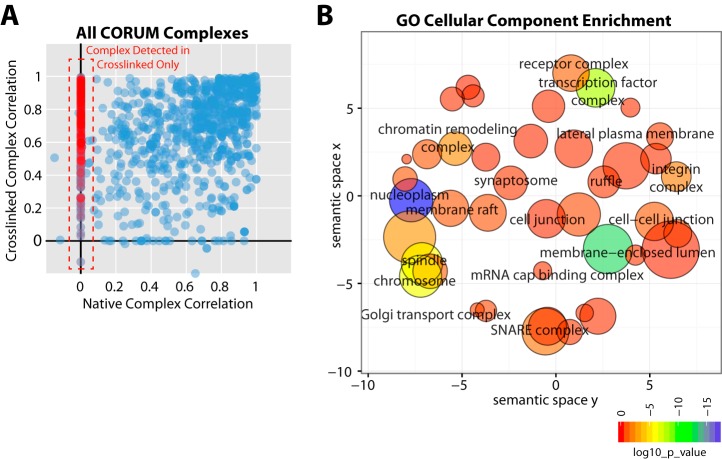
**CORUM-based systematic comparison between native and crosslinked SEC workflows for protein complex analysis.** CORUM complexes were included in the analysis if more than 2 protein components were detected in either the native, or crosslinked data sets. *A*, The median Pearson correlation calculated from all protein profile pairs for a single CORUM complex in either the previous native protein complex extraction (6), or the *in vivo* crosslinked and denatured extract from this study, are plotted as blue circles. CORUM complexes detected only in the crosslinked and denatured extracts (red circles inside dashed red box) are also shown. *B*, Analysis of enriched GO terms (cellular component) within the proteins belonging to complexes detected only from crosslinked and denatured extract. A significance threshold of EASE score < 0.05 from the DAVID database was used (49). Enriched GO terms were plotted using the REVIGO suite (50). The x and y axes indicate semantic space used to group GO terms of related cellular components, closer bubbles are more related. The color of each bubble indicates the EASE score (log10 transformed) for each GO term *versus* the whole human proteome. The size increases with increasing numbers of proteins associated with that term and the color changes from red to blue with increasing log10 (*p* value).

To facilitate a more systematic comparison between the native and crosslinked data sets, we used the CORUM protein complex database and identified all CORUM complexes detected by two or more protein components in either the native, or crosslinked data set. These analyses were based on identified proteins from all fractions and for each of these complexes (1,206 of 2,867 complexes in the CORUM database) the median Pearson correlation coefficient was calculated between all possible protein components in each data set. For each complex the median correlations were plotted (Fig. 6*A* and supplemental Table S3), which revealed that most of the detected complexes in U2OS cells had median correlations in both data sets >0.5. In addition, a subset of the CORUM complexes were only detected in the crosslinked data set (Fig. 6*A*, red circles). When the proteins present in the crosslinked-only complexes were analyzed for gene ontology cellular component enrichment using DAVID (49) and plotted using the REVIGO tool (50), we observed clear enrichment in integral membrane and membrane associated localizations (Fig. 6*B*). We also performed the same correlation analysis using complexes predicted by a recent PCP study (10), which generated a similar result to the CORUM-based analysis (supplemental Table S4).

**Fig. 7.**
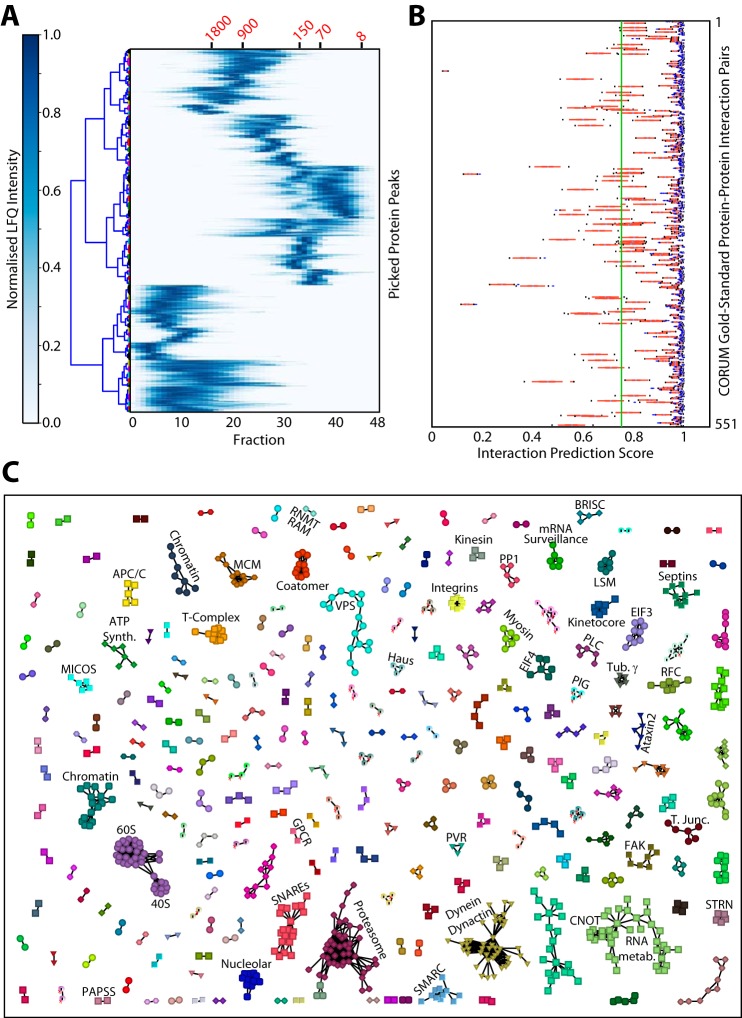
**Machine learning-based protein complex prediction from the *in vivo* crosslinked data set.** *A*, Heatmap showing the mean normalized LFQ intensity profile for the 5,336 protein peaks detected from the crosslinked data set profiles after filtering to remove void, dimer and monomer peaks. The elution points for molecular weight standards are shown (in kDa) in red text at the top of the plot. The similarity tree is shown to group similar peaks for visualization. *B*, Machine learning interaction predictor score distributions for all 551 true positive interaction peak pairs from the CORUM database. Red line shows the score distribution between the 25^th^ and 75^th^ percentiles. Black dots mark the 95% confidence intervals. The vertical green line across the plot marks the 0.75 interaction predictor score threshold, which was the minimum score allowed for the entire data set in the subsequent ClusterONE analysis. *C*, Protein complexes predicted by ClusterONE, visualized as a network map using VisANT (53). Colors are chosen randomly for each complex. Some of the known protein complex clusters are annotated with their name or abbreviation. 40S, small ribosomal subunit; 60S large ribosomal subunit; APC/C, anaphase promoting complex; BRISC, Brcc36-containing isopeptidase complex; CNOT, CCR4-NOT transcription complex; EIF3, eukaryotic translation initiation factor 3 complex; EIF4, eukaryotic translation initiation factor 4 complex; FAK, focal adhesion complex; GPCR, G-protein coupled receptor; MCM, minichromosome maintenance protein complex; MICOS, mitochondrial contact site complex; PAPSS, Bifunctional 3′-phosphoadenosine 5′-phosphosulfate synthase; PIG, GPI transamidase complex; PLC, phospholipase C; PP1, protein phosphatase 1; PVR, polio virus receptor; RFC, replication factor C complex; RNMT, mRNA cap guanine-N7 methyltransferase; RAM, RNMT-activating mini protein; SMARC, SWI/SNF complex; SNAREs, soluble NSF (n-ethylmaleimide sensitive factor) attachment protein receptors; STRN, striatins; VPS, vaculolar protein sorting proteins.

Given that we have observed many crosslinked protein profiles displaying multiple peaks, we have also performed an advanced protein-protein interaction prediction analysis similar to that described previously (9, 10). The first step in this analysis was picking individual peaks from within each profile in the crosslinked data set, which yielded 8,620 separate protein peaks (supplemental Table S5). These peaks were filtered (for an example see supplemental Fig. S4) to remove those eluting within the void fractions and in size ranges corresponding either to the protein's monomeric, or dimeric, molecular weights (supplemental Table S6). These peaks were plotted as a heatmap to provide an overview (Fig. 7*A*).

In the next step we used a machine learning approach similar to the one described previously (9, 10) to generate a single interaction prediction score, which combined multiple protein peak attributes with information retrieved from protein interaction databases. The prediction system (described in methods) was trained using >90 gold-standard complexes from the CORUM database, which were split into 551 true positive interaction pairs (supplemental Table S7). After training of the predictors these true positive pairs had an average score distribution >0.75. Therefore, we used a prediction score threshold for positive interactions in the whole crosslinked data set of >0.75 (supplemental Table S8). We supplied these positive interactions to the ClusterOne algorithm (30) as described previously (9, 10), which aggregated these interaction pairs into multi-protein complexes. This resulted in the prediction of 475 protein complexes (Fig. 7*C*), including 63 membrane complexes and many other previously known complexes, but also featuring multiple predictions of novel protein interactions (supplemental Table S9).

To facilitate the sharing of these data with the biomedical research community, in addition to depositing the raw MS data files via Proteome Exchange, we have designed a web-based interface for our EPD database (peptracker.com/epd) (51), which can display a direct comparison of SEC elution curves from different protein groups. In addition, the basic clustering data from our analysis is shown in heatmap form and these coclustered proteins can be easily used as suggestions for comparing the overlap in SEC elution profiles. We have also included a convenient link to the STRING database, such that any nodes linked to the protein of interest by STRING can be easily overlaid. The predicted molecular weight of the monomer of the protein of interest is also shown, to allow comparison with the approximate sizes indicated on the SEC profile plot. Together, these tools allow open access for any researcher to compare SEC elution profiles of any proteins detected in the current U2OS cell data sets.

## DISCUSSION

In this study we have shown that by combining *in vivo* protein crosslinking, using formaldehyde, with denaturing extraction conditions, the methodology for global analysis of protein complexes by MS-based protein correlation profiling can be significantly improved. In particular, this approach greatly enhances the recovery and detection of integral membrane and membrane associated protein complexes, along with other forms of protein complexes that bind tightly other cell substructures and hence are poorly represented in native cell extracts. Thus, we show in an analysis of human U2OS cells that using the *in vivo* crosslinking approach it is now possible to resolve and characterize many forms of endogenous protein complexes that are not detected when soluble complexes from native U2OS cell extracts are analyzed (6).

We have incorporated the entire U2OS cell data set of endogenous protein complexes into a convenient, searchable online database, the “Encyclopedia of Proteome Dynamics,” (http://www.peptracker.com/epd/), providing open access to explore and display the data for any proteins of interest. The data are provided in a web-based graphical interface that allows for each of the thousands of proteins that were detected to be plotted, showing the mean and standard deviation of three sets of biological replicates. Profiles can be overlaid for either the coclustered proteins displayed, or any other proteins detected in the data set. This provides a powerful tool that can be combined with other complementary information, such as data from affinity purification experiments, providing useful predictions of potential protein-protein interactions to help prioritise further functional studies. In addition, all of the raw MS files used to generate the PCP data presented in this study have been deposited with the ProteomeXchange Consortium (http://proteomecentral.proteomexchange.org) via the PRIDE partner repository and are available for download (see Methods).

The analysis of membrane-associated protein complexes and membrane protein interactions, either through affinity based approaches, such as immunoprecipitation/affinity tag pull-down, or using protein correlation profiling methods, as in this study, has always been complicated by the need to extract the proteins from the lipid bilayer in a soluble form and preferably without large micelle formation. While specialized detergents have been developed to facilitate this extraction, each protein complex will have a different extraction efficiency and vulnerability for breaking its interactions with each detergent type. In addition, many membrane proteins are either heavily glycosylated, or have few regions able to yield LC-MS-compatible peptides, both of which contribute to the difficulty of their analysis.

To mitigate these issues, while aiming at proteome-wide coverage, we have used the approach of covalently locking together interacting proteins prior to cell lysis, using *in vivo* crosslinking with formaldehyde. This allows the use of denaturing extraction in SDS, which efficiently solubilizes most complexes and keeps them in solution for the duration of the analysis. It is also possible to incorporate into the workflow different crosslinking agents, such as hetero-bifunctional reagents containing both NHS-esters (lysine reactive) and diazirine groups (photoactivatable, react with any amino acid), to enhance membrane protein crosslinking. For example, diazirine crosslinkers can react with the many hydrophobic amino acids that will be prevalent in membrane protein complexes. By optimization of crosslinker concentration and/or type we believe that any type of complex and/or cell type may be analyzed using the crosslinking methodology described here.

One consequence of using SDS for extraction, however, is that the proteins within each complex become denatured, thereby increasing the overall size (cross-sectional area) of the structure. For size exclusion chromatography, this limits the maximum protein complex size that can be resolved for a given SEC column, with larger complexes moving into the void volume. One other possible consequence of this method is the production of complexes with different extents of either denaturation, or crosslinking, which will lead to peak broadening. To maximize coverage of protein complexes, one solution to this problem is to use SEC columns with larger pore sizes that can hence resolve larger complexes. An alternative is to use detergents such as CHAPS, which are effective at solubilizing membrane protein complexes, but without entirely denaturing all the component proteins (52), thereby effectively reducing both complex size and peak broadening.

The global analysis of protein complexes using native approaches still has its benefits, including the ease of analysis of soluble protein complexes and the ability to exploit either enzymatic, or affinity-based separations. However, as we have shown here, many membrane protein complexes and weakly bound complexes either cannot be detected, or would require specialized optimization of conditions to allow detection from native extracts. In contrast, the crosslinking and denaturing extraction method described here is more efficient and largely eliminates the need for specialized optimization, apart from choosing the cross linker type and concentration. However, the crosslinking method may lead to broader peak profiles as we observed with the SEC-based separation used here for some types of complexes. These include complexes that are bound to either RNA/DNA, or substrate proteins, such as ribosomes/histones and chaperones respectively. This occurs because these complexes will be covalently crosslinked to a wide variety of hybrid structures with a large size range. Under native conditions, many of these complexes may either dissociate or be degraded and therefore yield narrower peaks that may be better clustered.

The strategies used here (clustering and machine learning) to analyze the coelution profiles of proteins result in predictions as to which proteins may exist in common complexes within cells, but do not prove that direct interactions occur between the cofractionating proteins. In this regard it may be possible in future to combine the protein correlation profiling approach with the use of crosslinking strategies that facilitate direct mapping of protein-protein crosslinks by MS analysis of material in each SEC fraction. Otherwise, the identity of protein complexes predicted by the SEC analysis can be confirmed either using additional information, for example from the existing literature and databases (as facilitated by the links provided in the EPD), or via further experimental analysis, or both.

The use of combined *in vivo* crosslinking-SEC-MS methodology, which allows systematic analysis of large numbers of endogenous, untagged proteins in cells and tissues, opens up many opportunities for future system-wide analyses of endogenous protein complexes and their differential responses to biological stimuli and regulatory mechanisms. We have shown here that by using formaldehyde crosslinking *in vivo*, chromatography-MS-based approaches for characterizing protein-protein interactions can now be extended to survey a more comprehensive set of cellular complexes in parallel. In particular, using crosslinking helps avoid the bias that limits detection of complexes not efficiently extracted and/or resolved in soluble, native extracts. Crosslinking also allows detection of protein subunits otherwise too weakly bound to be recovered from cell extracts. Although we have focused the present study on analysis of human U2OS cells, the method can readily be applied to other cell lines, model organisms and tissue samples. For example, *in vivo* crosslinking can be combined with studies of protein complexes in whole organisms by perfusion of formaldehyde into mouse tissue (19). There is also considerable scope to extend the approach further, for example by using alternative crosslinking agents and/or by using alternative chromatographic separation methods that are orthogonal to SEC (9, 10).

With all of these variations, we anticipate that the *in vivo* crosslinking-chromatography-MS approach can have widespread future applications for the global characterization and mechanistic studies of protein complexes and their dynamics throughout cell biology.

## Supplementary Material

Supplemental Data

## Footnotes

^1^ The abbreviations used are:

LC-MS/MS: liquid chromatography-tandem mass spectrometry
SEC: size exclusion chromatography
TARS: threonine tRNA ligase
XPNPEP1: Xaa-Pro aminopeptidase.
