# Supplementary material for: Global Membrane Protein Interactome Analysis using In vivo Crosslinking and Mass Spectrometry-based Protein Correlation Profiling
Source: Mol Cell Proteomics. 2016 Apr 25;15(7):2476–90. doi: 10.1074/mcp.O115.055467 (PMC4937518; doi:10.1074/mcp.O115.055467)
Supplement: Supplemental Data [file supp_15_7_2476__index.html]

Global Membrane Protein Interactome Analysis using In vivo Crosslinking and MS-based Protein Correlation Profiling — Global Membrane Protein Interactome Analysis using In vivo Crosslinking and Mass Spectrometry-based Protein Correlation Profiling — Crosslinking Enables Global Protein Interaction Analysis — Supplemental Data 

# Global Membrane Protein Interactome Analysis using *In vivo* Crosslinking and Mass Spectrometry-based Protein Correlation Profiling

## Supplemental Data

- Supplementary Figures (.pdf, 1.0 MB) - Supplementary Figures
- Supplementary Table 1 (.zip, 571.8 MB) - Maxquant output with all peptide-level data.
- Supplementary Table 2 (.xlsx, 62.0 MB) - All protein-level data from the native and crosslinked complex analysis.
- Supplementary Table 3 (.xlsx, 107 KB) - Native versus Crosslinked Comparison using CORUM Complexes
- Supplementary Table 4 (.xlsx, 61 KB) - Native versus Crosslinked Comparison using Wan et. al. Complexes
- Supplementary Table 5 (.xlsx, 3.5 MB) - All Detected Protein Peaks of Crosslinked Complexes
- Supplementary Table 6 (.xlsx, 2.1 MB) - Protein Peaks used for Machine Learning of Crosslinked Complexes
- Supplementary Table 7 (.xlsx, 17 KB) - Gold Standard CORUM Complexes
- Supplementary Table 8 (.xlsx, 5.8 MB) - Protein Peak Interaction Pairs with Prediction Score >0.75 of Crosslinked Complexes
- Supplementary Table 9 (.xlsx, 38 KB) - Crosslinked Protein Complexes Detected by Machine Learning Algorithm
