# Supplementary material for: Global Membrane Protein Interactome Analysis using In vivo Crosslinking and Mass Spectrometry-based Protein Correlation Profiling
Source: Mol Cell Proteomics. 2016 Apr 25;15(7):2476–90. doi: 10.1074/mcp.O115.055467 (PMC4937518; doi:10.1074/mcp.O115.055467)

SUPPLEMENTARY FIGURE LEGENDS

Supplementary Figure 1. Stabilization of protein interactions by *in vivo* crosslinking enables separation of protein complexes. a) UV absorbance chromatograms for the denaturing SEC separation on the SEC1000 column of either non-crosslinked, or crosslinked U2OS cells. b) SDS-PAGE and SYPRO Ruby staining of fractions collected from denaturing SEC separation on a SEC1000 column of either non-crosslinked, or crosslinked U2OS cells.

Supplementary Figure 2. Comparison between native and crosslinked SEC workflows for soluble protein complex analysis. a) Minichromosome maintenance complex, b) mitochondrial 28S ribosomal subunit, c) U2-snRNP complex, d) HSP90 chaperone complex. For each complex the upper grey panel shows the protein profiles from the previous native SEC method (6) with 40 fractions total and the lower grey panel shows the profiles from the *in vivo* crosslinked and denatured extract from this study with 48 fractions total. The x-axis shows the fraction number and the y-axis shows the normalized LFQ intensity. The line is the mean profile and the surrounding ribbon shows the standard deviation across the three biological replicates (n=3). The elution points for molecular weight standards are shown in red text under each axis in kDa.

Supplementary Figure 3. Comparison between native and crosslinked SEC workflows for the analysis of mitochondrial respiratory complex I interactions. Heatmaps show the mean normalized LFQ intensity profiles for all detected protein components of mitochondrial respiratory complex I (n=3). The upper heatmap shows the protein profiles from the previous native SEC method (6) with 40 fractions total and the lower heatmap shows the profiles from the *in vivo* crosslinked and denatured extract from this study with 48 fractions total. The x-axis shows the fraction number and the y-axis lists all the proteins plotted with a similarity tree for each dataset. The elution points for molecular weight standards are shown in red text under each x-axis in kDa.

Supplementary Figure 4. Machine learning-based protein complex prediction from the *in vivo* crosslinked dataset. a) Regions of the mean protein profile used for machine learning interaction

predictor for the *in vivo* crosslinked dataset. The regions are protein-specific and the diagram shows those calculated for isoform 2 of extended synaptotagmin-2. The x-axis shows the fraction number and the y-axis shows the normalized LFQ intensity. The line is the mean profile across the three biological replicates (n=3). The elution points for molecular weight standards are shown in red text at the top of the plot in kDa. The fraction regions used to delineate void, dimer and monomer/degraded peaks that are not used for interaction analysis are shown below the plot in red. The fraction region used to delineate peaks for interactions analysis is shown below the plot in green. b) Density plot of the distribution of the number of subunits for each of the 475 identified protein complexes. Most complexes were predicted to contain 2-3 protein subunits, with a much smaller number complexes containing 10-25 subunits.

Supplementary Figure 1

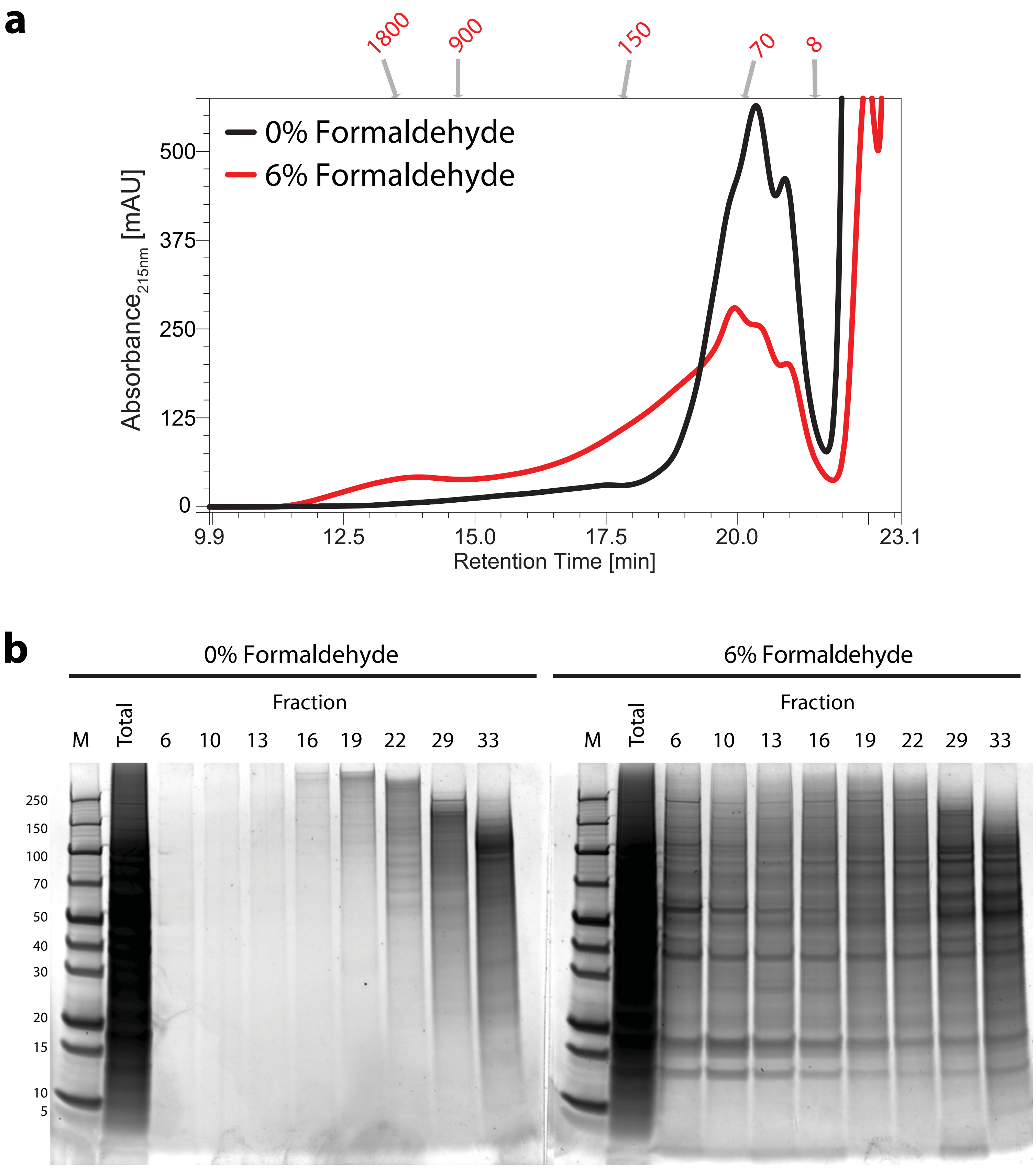

Supplementary Figure 2

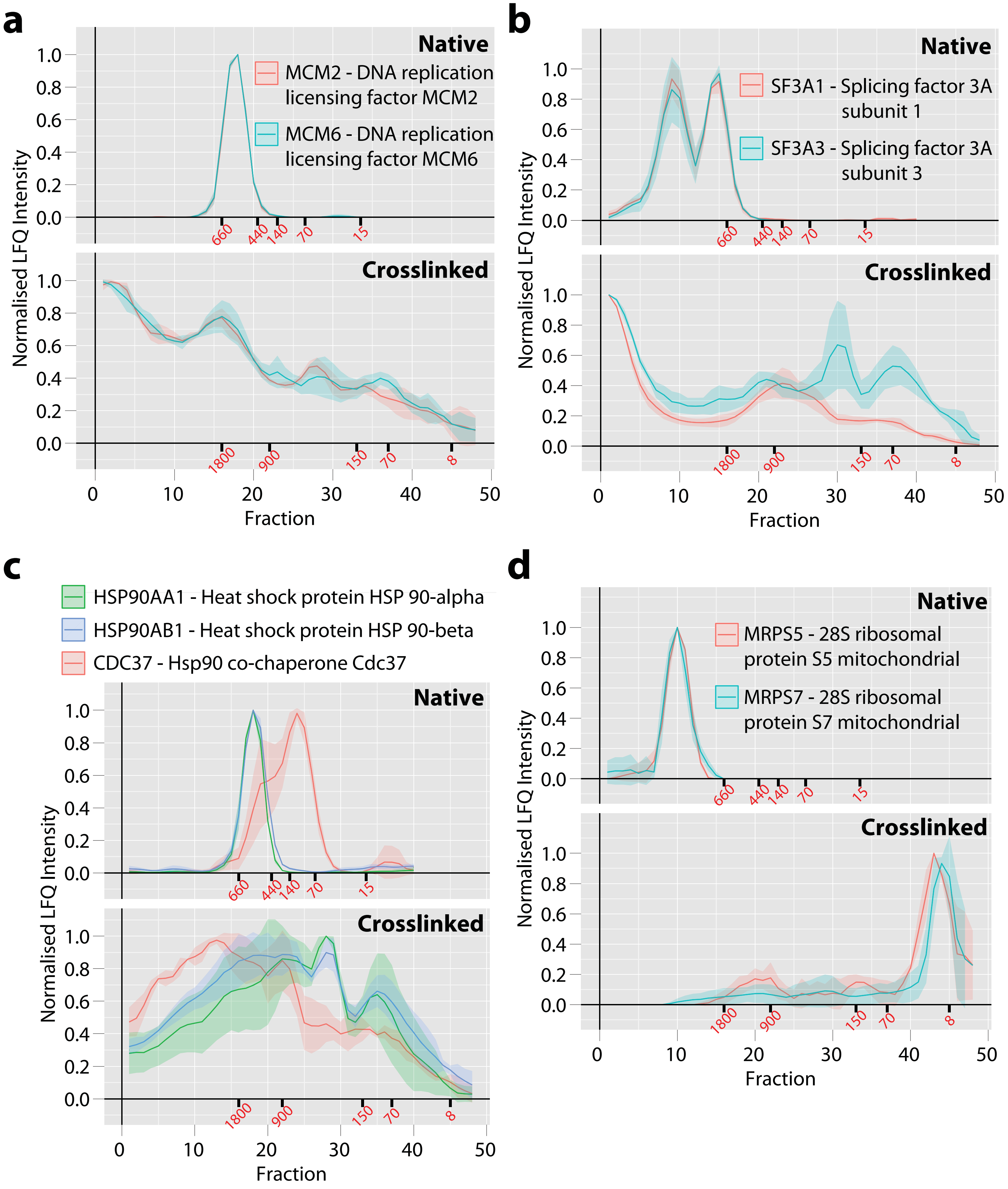

Supplementary Figure 3

Mitochondrial Respiratory Chain - Complex I

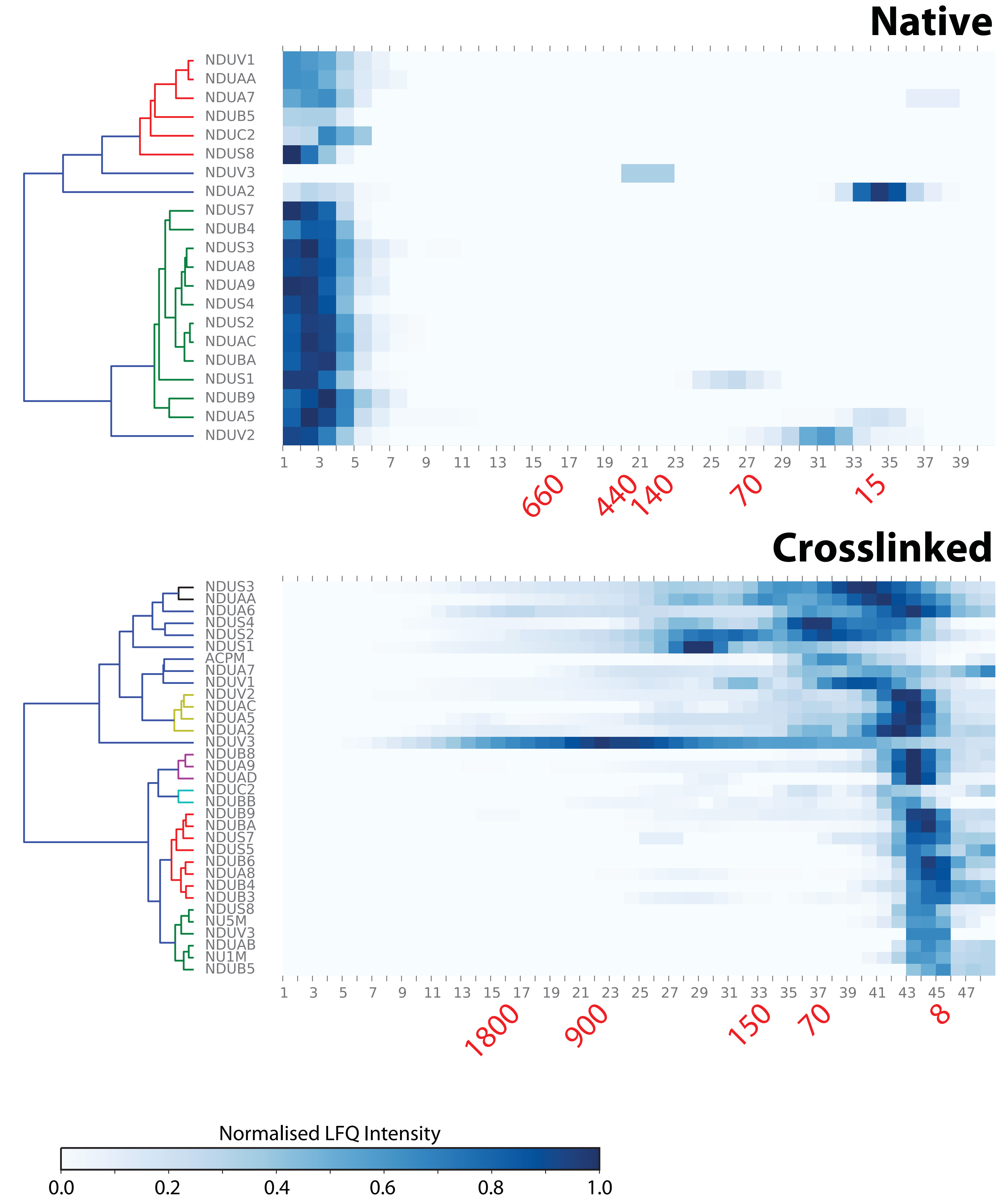

Supplementary Figure 4

a

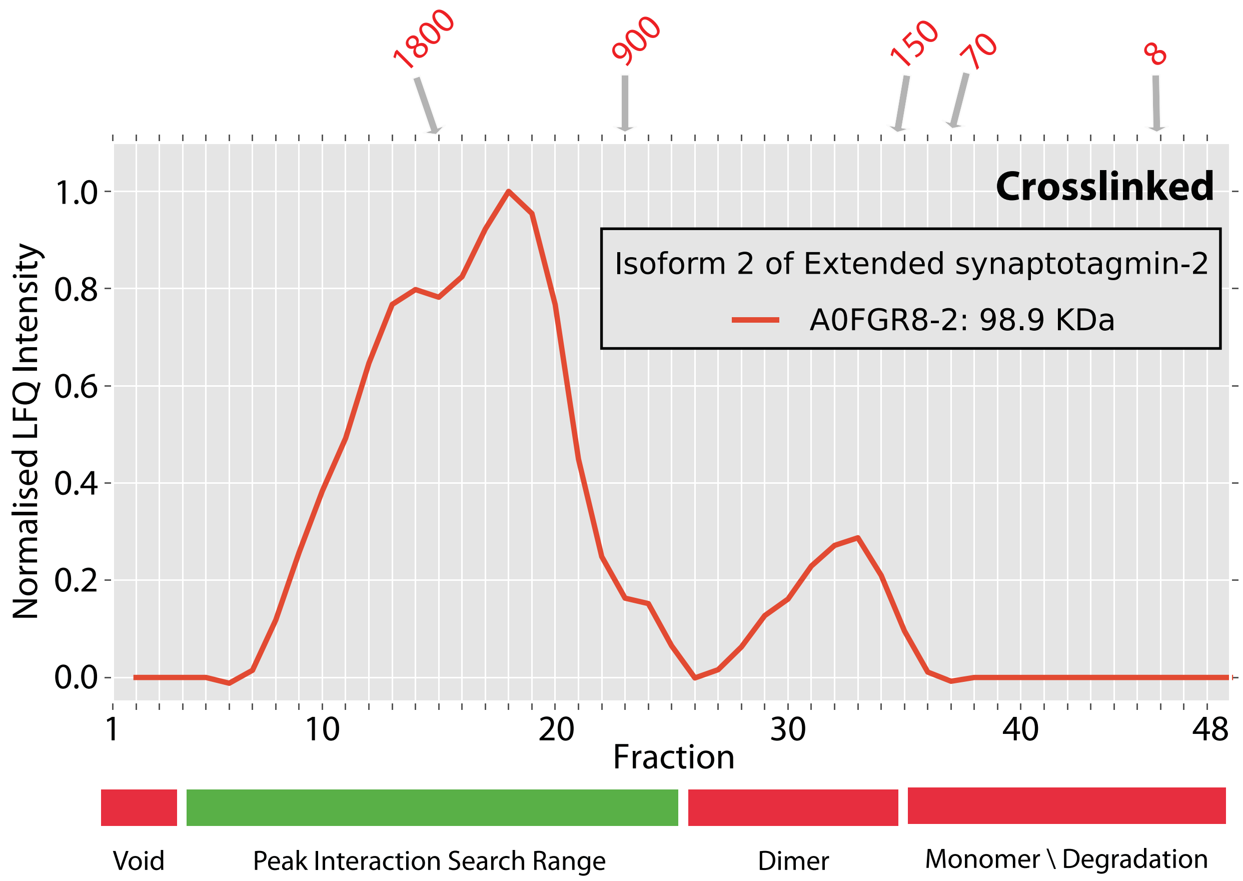

b

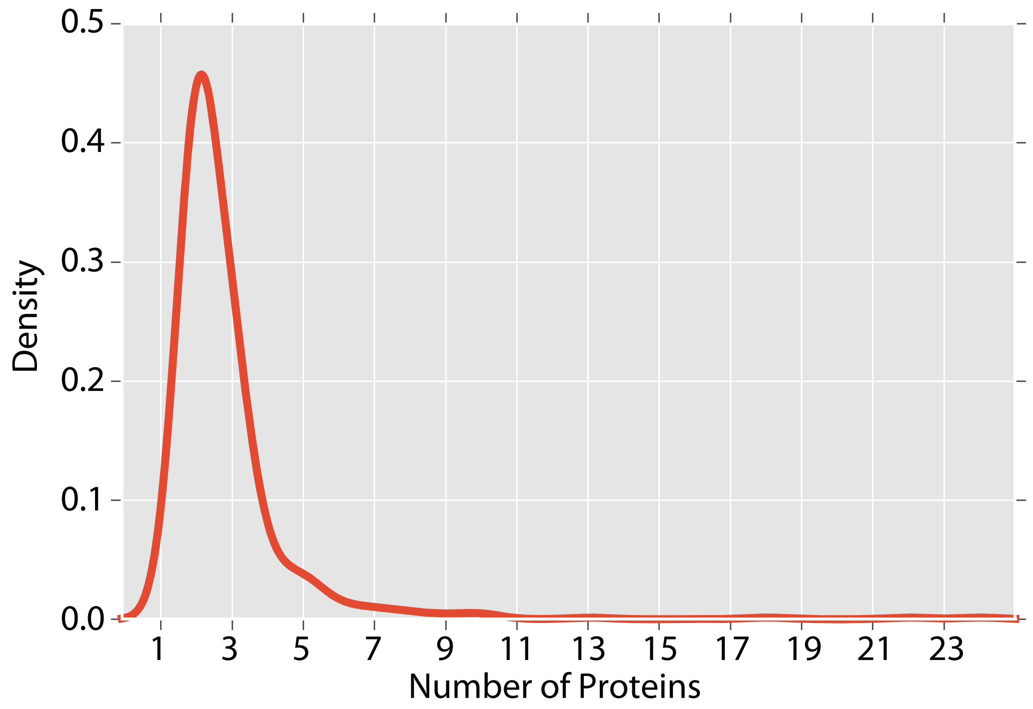

Supplement: Supplemental Data [file 10.1074_O115.055467_mcp.O115.055467-1.pdf]
